# Opinion: Endothelial Cells - Macrophage-Like Gatekeepers?

**DOI:** 10.3389/fimmu.2022.902945

**Published:** 2022-05-10

**Authors:** Amanda J. Stolarz, Shengyu Mu, Huiliang Zhang, Abdelrahman Y. Fouda, Nancy J. Rusch, Zufeng Ding

**Affiliations:** ^1^ Department of Pharmaceutical Sciences, College of Pharmacy, College of Medicine, University of Arkansas for Medical Sciences, Little Rock, AR, United States; ^2^ Department of Pharmacology and Toxicology, College of Medicine, University of Arkansas for Medical Sciences, Little Rock, AR, United States

**Keywords:** endothelial cells, macrophages, phagocytosis, autophagy, innate immunity

## Introduction

Endothelial cells are known to form a single-cell layer of endothelium that plays an important role in cardiovascular homeostasis by regulating blood fluidity, fibrinolysis, vascular tone and permeability, angiogenesis, monocyte adhesion, and platelet aggregation (1). Recent findings have demonstrated similarities between endothelial cells (ECs) and macrophages (2–5). Whereas they generally are not viewed as classic immune cells, ECs express a variety of innate immune receptors including Toll-like receptors (TLRs) and NOD-like receptors (NLRs), which activate intracellular inflammatory pathways mediated by nuclear factor kappa B (NF-κB) and the mitogen-activated protein kinases (MAPKs) (2, 6, 7). More interestingly, ECs and macrophages share evolutionarily conserved defense mechanisms such as phagocytosis and autophagy (8, 9). Macrophages are considered professional phagocytes and are highly specialized in the detection and removal of pathogens, apoptotic cells, and cell debris (3, 10). Additionally, macrophages are classical immune cells and play important roles in the innate immune system (11). Based on recent findings and considering the similar functions and mechanistic events shared by ECs and macrophages, we explore the opinion that ECs should be considered macrophage-like gatekeepers.

## ECs and Phagocytosis

Phagocytosis is a process by which a cell engulfs a large particle (≥ 0.5 μm), which may include foreign substances, microorganisms, and apoptotic cells. Thus, phagocytosis is a major mechanism in innate immune defense used to remove pathogens and cell debris. Professional phagocytes include monocytes, macrophages, neutrophils, tissue dendritic cells and mast cells. Professional phagocytes are the key players in removing dead cells; however, at certain time points and locations, the recruitment of professional phagocytes to the lesion site may be delayed or even absent (12). In these circumstances, non-professional phagocytes, including epithelial cells, endothelial cells, fibroblasts, and mesenchymal cells may fulfill this role. Increasing evidence suggests that ECs also are capable of performing phagocytosis of dead cells in the blood or neighboring microenvironment. For example, a recent study by Zhou et al. demonstrated that, *in vivo*, microvascular ECs engulf myelin debris during spinal cord injury and promote recruitment of macrophages and development of fibrosis after neural injury (10). *In vitro*, primary mouse brain microvascular endothelial cells (BMECs) were grown on Matrigel and uptake of myelin debris was compared with bone marrow-derived macrophages. BMECs showed the ability to uptake myelin debris at 24 to 96 hours after injury compared with bone marrow-derived macrophages that showed rapid myelin engulfment as early as 1 to 3 hours. This study provided initial evidence that ECs can act as non-professional phagocytes. Zhou et al. further revealed that IgG opsonization is required for effective engulfment of myelin debris by BMECs and delivery to the autophagy-lysosome pathway for intracellular degradation (10).

Similar to BMECs, vascular ECs were shown earlier to internalize large fibrin or cholesterol clots that are subsequently released into the underlying parenchyma (13). This process may play an important role in the development of cardiovascular diseases, such as atherosclerosis, myocardial infarction, and vascular aging. Dini et al. reported that 60 minutes after co-incubating liver ECs with apoptotic cells, the majority of apoptotic bodies were seen inside EC phagosomes and only a few remained at the cell surface (3). Another study by Xie et al. showed that apoptotic bodies were incorporated into primary human umbilical vein endothelial cells (HUVECs) followed by overexpression of α-integrins within 1.5 hours. At 3 hours, these apoptotic bodies were fully digested within the ECs as nearly all phagocytosed intracellular materials had disappeared (9). Rengarajan et al. showed that cultured primary HUVECs use a phagocytosis-like process to internalize both pathogenic and non-pathogenic Listeria bacteria (13). ECs may contribute to pathogen removal by killing internalized *Rickettsiae* in a cytokine-activated hydrogen peroxide- or nitric oxide-dependent manner (14). It is speculated that endothelial phagocytosis-like uptake is a surveillance strategy to remove particles from the bloodstream, particularly in cases of macrophage injury, or at sites at which macrophages have limited access. More interestingly, phagocytosis-like uptake by ECs also may recruit immune cells specifically to vulnerable sites in the vasculature to limit pathogen dissemination (13). For example, abundant senescent neutrophils may become procoagulant when undergoing apoptosis and may contribute to thrombosis or inflammation. A study by Gao et al. showed that co-culture of aged neutrophils with ECs resulted in phagocytosis of the neutrophils and prolonged coagulation time (5). Therefore, the ability of ECs to clear senescent neutrophils by phagocytosis may limit the procoagulant and/or inflammatory response. In other non-cardiovascular diseases such as acute promyelocytic leukemia (APL), *in vitro* studies suggest that phagocytosis of APL cells by macrophages and ECs may play a role in the prevention of APL coagulation disorder (9). In this regard, it has been reported that ECs can engulf intravascular thrombi in the brain and other organs and translocate them into the perivascular space in a process termed ‘angiophagy’. However, the process takes several days and may be associated with reduced tissue plasminogen activator-mediated fibrinolysis in the early hours due to EC lamellipodia surrounding the emboli (15). Similar to this phenomenon, uptake of erythrocytes by ECs has been reported in response to microbleeds. This process of erythrophagocytosis, a well-described feature of macrophages that enables removal of aged or damaged red blood cells, is believed to be conducted by brain and peripheral endothelium (4, 16). Collectively these studies provide persuasive evidence that ECs are capable of phagocytosis.

## ECs and Autophagy

In contrast to phagocytosis that refers to cell internalization of extracellular materials, autophagy denotes the process by which an intracellular degradation system removes dysfunctional organelles, abnormal aggregated proteins, lipid accumulation, and infecting pathogens. Autophagy as a physiological function is a protective mechanism that allows recycling of defective organelles and proteins to maintain cellular homeostasis. As the inner lining of all subvascular compartments, ECs supply nutrients and oxygen to the parenchymal tissue to maintain tissue homeostasis (8). Most ECs are in a healthy, quiescent state; however, ECs can dynamically respond to microenvironmental changes or stimuli that may include oxidative stress, unfolded proteins, low oxygen or nutrient availability. Among these stimuli, hypoxia and nutrient deprivation represent common features of the vascular microenvironment that may activate ECs. In such cases, after vessel reperfusion and restoration of physiological levels of oxygen and nutrients, ECs can return to the quiescent state. Autophagy is associated with the unfolded protein response (UPR) and the mammalian target of rapamycin (mTOR), which regulate ECs adaptation and survival. Intrinsic autophagy modulates the response of ECs to various metabolic stressors and has a fundamental role in nitric oxide production, angiogenesis, and hemostasis and thrombosis (17).

The importance of autophagy in ECs is increasingly recognized but only explored in more detail in recent years. In the setting of atherosclerosis, ECs become chronically activated through a combination of turbulent blood flow, lipid accumulation and exposure to inflammatory mediators (1). Atherosclerotic plaques tend to develop preferentially in areas of the vasculature such as the aortic arch and bifurcation that are exposed to low and disturbed shear stress. Vion et al. demonstrated that defective autophagy in ECs not only restricts endothelial alignment with the direction of blood flow, but also promotes an inflammatory, apoptotic, and senescent phenotype (18). In this study, HUVECs were treated with bafilomycin A1, an inhibitor of autophagic flux, to evaluate the ratio of LC3II/LC3I, a widely used marker of autophagy flux in response to shear stress. The results showed that bafilomycin A1 increased the LC3II/LC3I ratio in HUVECs exposed to high shear stress for 6 hours, while bafilomycin A1 had no effect on the LC3II/LC3I ratio in HUVECs under low shear stress conditions. This indicates a functional autophagic flux in HUVECs exposed to high shear stress and a blockade of the fusion between autophagosomes and lysosomes in HUVECs exposed to low shear stress. Additional findings *in vivo* also supported a direct effect of endothelial autophagy on atherosclerotic plaque formation rather than an effect on systemic metabolic parameters (18). Reglero-Real et al. recently revealed a novel mechanism on how autophagy makes EC an immune cell (19). Autophagy regulated the remodeling of EC junctions and expression of key EC adhesion molecules, facilitating their intracellular trafficking and degradation (19). Therefore, it is reasonable to consider autophagy as a modulator of EC leukocyte trafficking machinery aimed at terminating physiological inflammation and immune response.

Defective autophagy in ECs also may be associated with diverse types of metabolic diseases such as type 2 diabetes. Endothelial to mesenchymal transition (EndMT) is a process by which ECs undergo a change in phenotype to resemble a mesenchymal cell, such as a myofibroblast or smooth muscle cell (20). Takagakia et al. reported that autophagy defects in ECs induced IL6 (interleukin 6)-dependent endothelial-to-mesenchymal transition (EndMT) and organ fibrosis with metabolic defects in mice (21). *In vitro* studies revealed that siRNA transfection of the autophagy related 5 (ATG5) gene in human microvascular endothelial cells (HMVECs) induced autophagy defects characterized by a decreased LC3II/LC3I ratio and SQSTM1/p62 accumulation (21). To simulate these conditions in the setting of clinical type 2 diabetes, this group generated an endothelial-specific ATG5 knockout using a diet-induced mouse model (ATG5 Endo) of obesity (21). Their results showed that ATG5 Endo displayed progressive EndMT and fibrosis, especially in the high fat diet condition, potentially implicating metabolic defects. These data demonstrated that autophagy in ECs is essential for the integrity of ECs and that defective autophagy in ECs may induce fibrosis, inflammation, and vascular defects.

## ECs and the Innate Immune System

**Figure 1:**
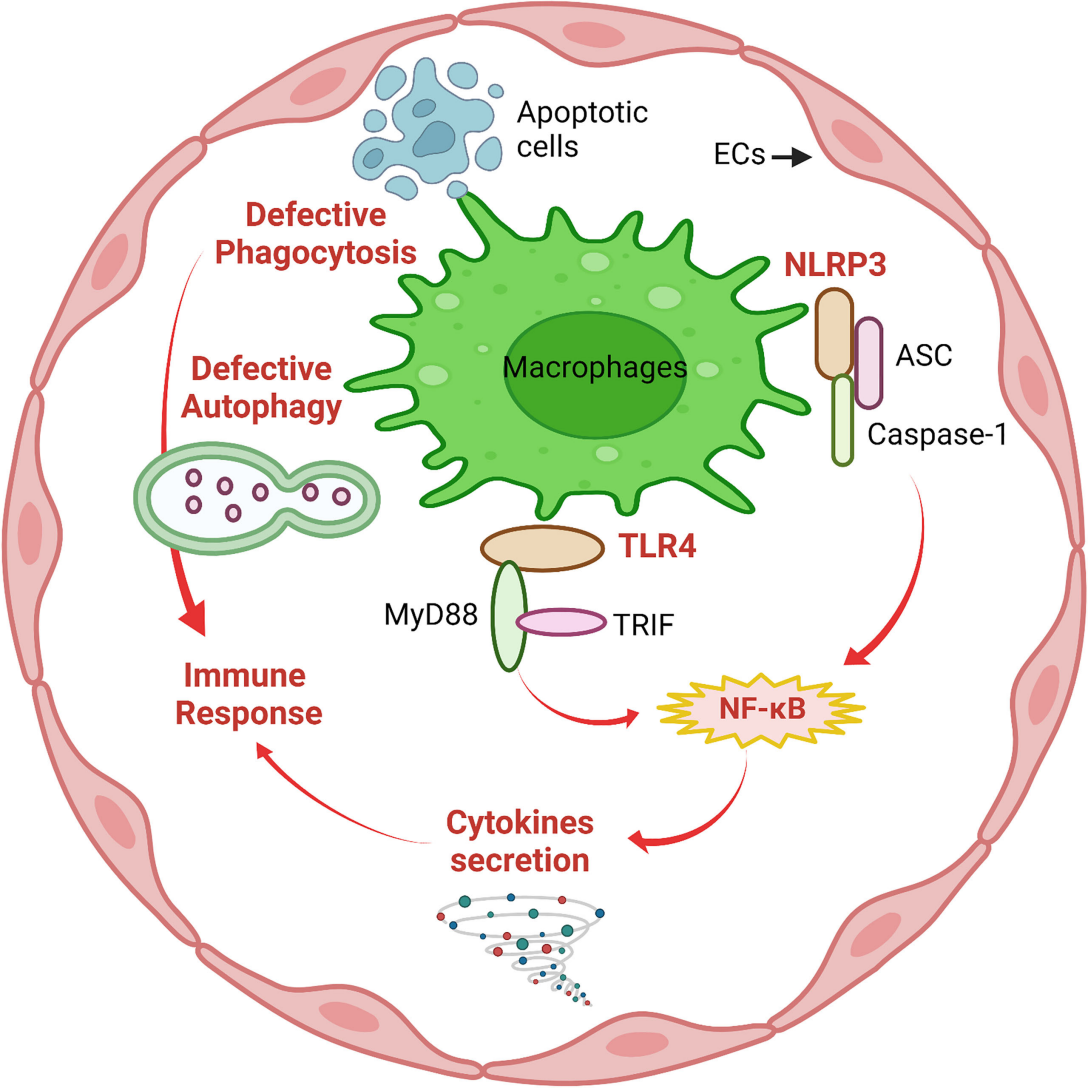
Macrophage-like functions in ECs. Similar to macrophages, defective phagocytosis and autophagy are implicated in deficient immune responses of ECs. The innate immune system in ECs involves activation of NLRP3 inflammasome and TLR4/MyD88/TRIF signaling followed by NF-κB-mediated secretion of pro-inflammatory cytokines to mediate the immune response. This figure was created with Biorender.com.

The innate immune system is the first line of defense against invaders such as viruses, bacteria, parasites and toxins, or it also senses wounds or trauma. Due to their location in the vessel lumen, ECs are one of the first cells to interact with invaders in the circulation. A growing body of evidence supports that EC recognition and response contribute to early innate immune system activation (11). Toll-like receptors (TLRs) and NOD-like receptors (NLRs) are members of the Pattern Recognition Receptor (PRR) family, which recognizes pathogen- associated molecular patterns (PAMPs) and plays a crucial role in the innate immune system by defending against infections (22). ECs can express both TLRs and NLRs as well as chemokine receptors. When incubated with lipopolysaccharide (LPS), a major component of Gram-negative bacteria cell walls, ECs produce an acute inflammatory response (23). Among TLRs, TLR4 acts as a specific pattern-recognition receptor for LPS and co-activates the myeloid differentiation primary response 88 (MyD88)-dependent and TIR-domain-containing adapter-inducing interferon-β (TRIF)-dependent pathways (24). TLR4 upregulation activates both nuclear factor-κB (NF-κB) and interferon (IFN) signaling pathways and induces the production of pro-inflammatory cytokines, such as interleukin-1 (IL-1) family, monocyte chemotactic protein-1 (MCP-1), and tumor necrosis factor-α (TNF-α) (7). The NOD-like receptor protein 3 (NLRP3) inflammasome is an intracellular multimeric complex that triggers the activation of cysteine-aspartic acid protease-1 (caspase-1) and the maturation of IL-1β and IL-18 (25). Both TLR4 signaling and NLRP3 inflammasome signaling are traditional pathways in response to the innate immune system in macrophages; however, a large body of evidence suggests that TLR4 signaling and NLRP3 inflammasome signaling also play critical roles in ECs. This concept has been reviewed well in earlier articles (2, 6, 26, 27) and is summarized in **Figure 1**.

## Conclusions and Perspectives

Vascular ECs act as gatekeepers and adopt a macrophage-like function in the innate immune system to protect underlying tissue from blood-borne toxins and pathogens. Nevertheless, the precise mechanisms of macrophage-like function in ECs that may include phagocytosis, autophagy and the innate immune response remain largely unknown. In recent years, an increasing body of evidence suggests that ECs function as macrophages in the clearance of apoptotic cells, cell debris and pathogens and in the recruitment of immune cells to vulnerable sites in the vasculature to limit pathogen dissemination. Insights into the macrophage-like function exhibited by ECs have been unveiled gradually in recent years, but there still are many holes in our knowledge of the signaling pathways regulating this process. For example, how do ECs sense apoptotic cells and pathogens and does this happen in all tissues or only in neighboring cells? What is the fate of an EC after uptake of apoptotic cells and pathogens? And what is the product of EC phagocytosis? The answers to these questions may help to clarify the macrophage-like physiological functions of ECs and reveal strategies for targeting the diverse roles of the endothelium to deter vascular disease.

## Author Contributions

ZD wrote the first draft of the manuscript. AS, SM, HZ, AF, and NR contributed sections of the manuscript. All authors contributed to the manuscript revision, read, and approved the submitted version.

## Funding

This work was supported by the National Institutes of Health, including National Institute on Aging [R21AG075548], and National Heart, Lung, and Blood Institute [R01HL162958 and R01HL146713]. National Institute of General Medical Sciences Centers of Biomedical Research Excellence the Center for Studies of Host Response to Cancer Therapy [P20GM109005]. The content is solely the responsibility of the authors and does not necessarily represent the official views of the NIH. The work was also partially supported by the UAMS Bronson Endowment Foundation Award, VCRI Equipment Grant and Shared Resources Voucher Award.

## Conflict of Interest

The authors declare that the research was conducted in the absence of any commercial or financial relationships that could be construed as a potential conflict of interest.

## Publisher’s Note

All claims expressed in this article are solely those of the authors and do not necessarily represent those of their affiliated organizations, or those of the publisher, the editors and the reviewers. Any product that may be evaluated in this article, or claim that may be made by its manufacturer, is not guaranteed or endorsed by the publisher.
